# Menstrual hygiene management amongst schoolgirls in the Rukungiri district of Uganda and the impact on their education: a cross-sectional study

**DOI:** 10.11604/pamj.2014.19.253.5313

**Published:** 2014-11-07

**Authors:** Robyn Boosey, Georgina Prestwich, Toity Deave

**Affiliations:** 1 Department of Sociology, Politics, and International Studies, University of Bristol, Bristol, UK; 2 Faculty of Medicine and Dentistry, University of Bristol, Bristol, UK; 3 Centre for Child and Adolescent Health, University of the West of England, Bristol, UK

**Keywords:** Menstruation, education, health, adolescents, low-income countries, middle-income countries

## Abstract

**Introduction:**

An increasing number of studies have found that girls in low-income settings miss or struggle at school during menstruation if they are unable to manage their menstrual hygiene effectively. This study explores the menstrual hygiene practices and knowledge of girls at rural government primary schools in the Rukungiri district in Uganda and assesses the extent to which poor menstrual hygiene management (MHM) affects their education.

**Methods:**

A self-administered questionnaire was completed by schoolgirls in six government-run primary schools in the Rukungiri district. Focus groups were held with girls from each school and semi-structured interviews were conducted with headteachers and female teachers from the participating schools. A toilet assessment was also conducted in each school.

**Results:**

One hundred and forty schoolgirls completed the questionnaire. The girls reported a lack of access to adequate resources, facilities and accurate information to manage their menstrual hygiene effectively at school. They reported that, as a result, during menstruation they often struggle at school or miss school. Eighty-six girls (61.7%) reported missing school each month for menstrual-related reasons (mean 1.64, range 0-10, SD. 1.84).

**Conclusion:**

It is common for girls who attend government-run primary schools in the Rukungiri district to miss school or struggle in lessons during menstruation because they do not have access to the resources, facilities, or information they need to manage for effective MHM. This is likely to have detrimental effects on their education and future prospects. A large-scale study is needed to explore the extent of this issue.

## Introduction

School dropout for girls in low-income settings increases when they reach puberty [1–3]. Previously overlooked menstrual-related concerns are increasingly recognised as factors that contribute to this [4–8]. However, research into the impact of unmet menstrual needs on girls’ education remains scarce. The dearth of studies about menstrual hygiene management (MHM) and the impact of unmet menstrual needs is particularly striking given the relevance of the topic to numerous fields, such as WASH (water, sanitation and hygiene), humanitarian relief, and human rights.

Menarche is an important milestone in a girl's transition to womanhood. However, menstruation can place significant obstacles in the way of girls’ access to health, education and future prospects if they are not equipped for effective MHM. Good MHM requires access to necessary resources (e.g. menstrual materials to absorb or collect menstrual blood, soap and water), facilities (e,g. private place to wash, change and dry re-usable menstrual materials, in addition to an adequate disposal system for menstrual materials), and education about MHM [9].

MHM amongst girls in rural government-run primary schools in Uganda is an under-examined area of research. Girls in this setting are unlikely to have access to what they need to manage their menstrual flow and are thus more at risk of absenteeism from school [10–14]. Girls have a human right to education and educating girls is a wise investment for development, producing ‘high and long-lasting returns’ for families, societies and subsequent generations [15]. Therefore, research into the impact of poor MHM on girls’ education is vital.

This study aims to assess and explore the extent to which schoolgirls in a low-income setting in south-west Uganda are able to manage their menstrual hygiene effectively and if this impacts on their education. We aim to contribute to the emerging literature on MHM and to identify key areas where MHM interventions are needed. Irise International is a key NGO working on MHM, focusing on East Africa. The organisation invited the researchers to undertake this research in Uganda and connected them with their local contacts in the Rukungiri district for this project.

## Methods

This cross-sectional study adopts a mixed methods approach, combining four sources of data [16]: self-administered questionnaires, focus group discussions (FGDs), semi-structured interviews with key informants and a toilet assessment. The study was conducted in six rural, government-run primary schools in the Rukungiri District of south-west Uganda between March-April 2013. The schools were recruited through the headteacher of a local private school. This headteacher, who was the organisation's main contact in the education sector in the locality, supported the researchers in identifying the closest government-run primary schools to the central research facilities in Kisiizi hospital. Six schools were identified and agreed to participate. Girls aged 13-16 years who had started menstruation were invited to take part. The girls who participated were old enough to attend secondary school, yet remained in the upper end of primary school because they were behind with their studies. One hundred and seventy-three girls in total participated in the self-administred questionnaire. Prior to the research we informed the schools of the selection criteria that respondents had to meet to participate. However, when analysing the results we discovered that thirty-three girls had still participated who did not meet this criteria. Their questionnaires were consequently excluded from the final results.

Data collection took place in a private classroom, a private outdoor location in the school compound, and the school toilets. Participants gave written consent before participating and confidentiality and anonymity were emphasised at every stage. The girls sat a seat apart from each other when completing the questionnaire and could not converse amongst themselves so that they would not influence each other's answers. Participation was voluntary and girls were allowed to withdraw from the research at any stage if they wished to, without having to give a reason. Due to the sensitive nature of menstruation, the researchers and translator were female. There were no male pupils or teachers present during the questionnaire completion, FDGs or interviews with female teachers. The FGDs and interviews were audio-recorded and then transcribed verbatim for analysis. Before conducting the research, the field researchers received formal training from Irise International by staff members who have experience of working in this area.

The anonymous, self-administered questionnaire had been piloted in Kenya [14], reviewed by the local translator in Uganda and amended before use to make it accessible for respondents. It included sections on general absenteeism, knowledge about menstruation, menstrual practices, menstrual-related absenteeism and income level. The questionnaires were used to collect quantitative data on the girls’ experiences of menstruation as they have been found to increase respondents’ willingness to share sensitive information on health issues [17]. Participants are also less likely to under or over-estimate sensitive health problems [18] and less likely to have acquiescence ‘yes-saying’ response bias compared to when they participated in interviews or focus groups [19].

The questionnaire was written in English, but was translated into Rukiga by a local translator. The girls could ask questions in Rukiga whilst completing the questionnaire and answer questions in Rukiga to ensure that language was not a barrier. Their answers were then translated into English to be analysed.

Once the questionnaires had been completed, we conducted FGDs with six to nine girls in each school. Senior female teachers selected the girls, choosing the eldest post-menarche girls who had completed the questionnaire as they were the most willing to take part. We employed participatory methods, which are research activities designed to engage participants in the research process as equal partners and to draw out their ‘voiced experiences’ [20]. These methods were used because they have been found to empower research participants and facilitate interaction between them. Given the power dynamics of the research process and the taboo nature of menstruation, this is important [5, 21]. Moreover, it is essential to consult girls affected by poor MHM to gain an accurate understanding of the problem and find effective solutions [5, 22].

Semi-structured in-depth interviews were conducted with the headteachers from each of the six schools (all of whom were male), senior female teachers from five schools, and a female teacher in one school because the senior female teacher was absent. We interviewed the head teachers to gain their perspective on the MHM of the schoolgirls, because they are the highest school authority and the senior women teachers because they are responsible for the girls’ welfare, reproductive education and for supporting the girls during menstruation. The interview with the female teacher provided an additional perspective from another important female figure in the girls’ lives.

We drew on the girls’ toilet assessment criteria from Pillitteri [12] and WaterAid MHM toolkits [23] to develop a toilet assessment survey. The survey asked about the toilets’ design, construction, operation, maintenance, privacy, access for girls with disabilities, water supply, and disposal system for sanitary products. In each school a researcher assessed the toilets for girls using this survey. To ensure that the toilets were observed in their normal state, the researcher asked permission to visit them once the girls had completed the questionnaires and FDGs, rather than asking in advance. This prevented the school from tidying up the toilets to present them in a better condition than usual for the research.

We employed SPSS 19™ to enter and analyse the data. Descriptive statistics and frequencies were used to examine the characteristics of the study population. Interviews and FGDs were analysed by thematic content analysis [24]. The transcripts were coded and classified by both field researchers (Robyn Boosey and Georgina Prestwich) and then recurrent themes that emerged were discussed. We obtained ethical approval from the Management Committee of Church of Uganda Kisiizi Hospital and the University of Bristol.

## Results

### Questionnaire

One hundred and seventy-three girls completed the questionnaire, of whom 33 (19.1%) did not match the selection criteria for the following reasons: 8 (4.6%) were not the right age, 23 (13.3%) had not started menstruation, and 2 (1.2%) did not state whether or not they had started their period. The final sample size was 140. The mean age for the girls in the study population was 14.45 (SD 0.908). Table 1 presents the respondents’ socio-economic characteristics.

**Table 1:** Key questionnaire results

Focus	Statement/ Question	Responses (n)			No. of participants that responded to question
		True	False	Don't know	
Socio-economic characteristics of respondents	Over the past year have you or your family ever gone without enough food to eat?	82	52	N/A	140
	Over the past year have you or your family ever gone without enough clean water for home use?	66	74	N/A	140
	Over the past year have you or your family ever gone without medicine or medical treatment?	100	39	N/A	139
	Over the past year have you or your family ever gone without enough fuel to cook your food?	74	66	N/A	140
	Over the past year have you or your family ever gone without school expenses for fees, uniforms or books?	109	31	N/A	140
	Over the past year have you or your family ever gone without a cash income?	99	40	N/A	139
Knowledge about menstruation	Menstruation is a disease	49	86	N/A	135
	Pregnant women menstruate	31	104	N/A	135
	Menstrual blood comes from the stomach where food is digested	37	96	N/A	133
	Menstrual blood comes from the womb	116	20	N/A	136
	Menstrual blood contains harmful substances	83	50	N/A	133
	Pain during menstruation means that someone is unhealthy	66	68	N/A	134
	It is harmful for a woman's body if she runs or dances during her period	66	69	N/A	135
Reasons reported for menstrual-related absenteeism	Fear of staining my clothes	82	56	N/A	138
	Afraid of others making fun of me	64	73	N/A	137
	Menstruation can cause pain	71	67	N/A	138
	Menstruation can cause discomfort from bloating or tiredness	75	61	N/A	136
	There isn't anywhere private for girls to wash and change at school	88	50	N/A	138
	There is nowhere to dispose of sanitary products in school	60	78	N/A	138
	I do not have sanitary pads	63	75	N/A	138
Access to disposable sanitary pads	Have you bought disposable sanitary pads from the shop in the last 6 months?	50	85	0	135
	Have you ever wanted to buy disposable sanitary pads from a shop but been unable to?	122	17	0	139
	I do not have enough money to buy disposable sanitary pads from a shop	85	39	14	138
	There are no disposable sanitary pads in the shop	47	62	27	136

**Figure 1:**
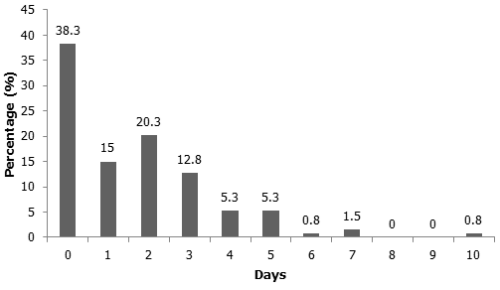
Bar graph showing number of days in a month girls missed due to menstruation (n = 133)

Respondents demonstrated poor knowledge of menstruation (Table 1). Menstrual-related absenteeism was prevalent amongst respondents 61.7% of whom miss school at least one day per month (mean 1.64, range 0-10, SD.1.84) (Figure 1). The main reason girls reported for menstrual-related absenteeism was the lack of a private place for them to wash and change at school (n = 88, 63.8%) (Table 1). This was followed by fear of staining their clothes (n = 82, 59.4%), discomfort from bloating and tiredness (n= 75, 55.1%), and pain (n-= 71, 51.4%) (Table 1). When asked if there were additional reasons for menstrual-related absenteeism not listed in the questionnaire, one girl stated that she was afraid of her menstrual cloth falling out if she was beaten at school.

**Figure 2:**
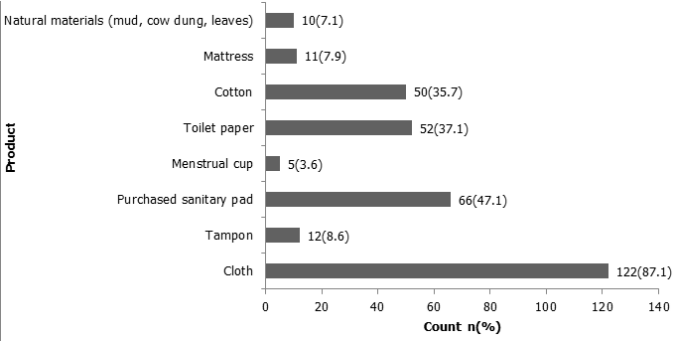
Bar graph showing girls’ reported normal menstrual product use (n = 140)

Figure 2 shows the different products that girls use to absorb menstrual blood. Even though girls report using mixed methods, the most common product used to absorb menstrual blood is cloth (n = 122, 87.1%) (Figure 2). Whilst 50 (37%) girls had bought disposable sanitary pads in the last 6 months, 122 (87.8%) had been prevented from buying disposable sanitary pads on at least one occasion because they could not afford them (61.6%) or because disposable pads were not available in local shops (34.6%) (Table 1).

### Toilet assessment

None of the girls’ school toilets assessed in any of the schools were adequate for good MHM (Table 2) due to their lack of cleanliness, light, access for girls with disabilities and soap and water, in addition to the poor ratio of toilets for the number of girls. The definitions used for the assessment of the toilets such as ‘sufficient’, ‘accessible’, and ‘clean’ are based on UNICEF's school WASH guidelines [7, 25]. ‘Sufficient light’ is understood to mean enough light to see what you are doing and if the facilities are clean when the cubicle door is closed. ‘Separate’ means that the girls’ toilets are in different blocks or designated areas separated from boys’ and teachers’ toilets by distance and/or some physical barrier like a wall.

**Table 2:** Toilet assessment results from the 6 participating schools

Question	School 1	School 2	School 3	School 4	School 5	School 6
What type of toilets are there?	Pit latrine	Pit latrine	Pit latrine	Pit latrine	Pit latrine	Pit latrine
Are there sufficient toilets for girls?	No	No	No	Yes	No	No
Are girls’ toilets easily accessible?	No	Yes	Yes	Yes	Yes	Yes
Is there soap available in the toilet facilities?	No	No	No	No	No	No
Are there vault cover slabs for the toilets?	No	No	No	No	No	No
Is there a mirror available in the toilets?	No	No	No	No	No	No
How clean are the girls’ latrines? (Clean/Somewhat clean/Not clean)	Not clean	Not clean	Not clean	Not clean	Not clean	Not clean
Do sufficient toilets work?	Yes	No	No	Yes	Yes	No
Are the paths to access the toilets well maintained?	Yes	No	Yes	No	No	No
Is there sufficient light in the toilets?	No	No	No	No	Yes	No
Is there an effective maintenance and cleaning routine in place for the toilets and MHM facilities?	No	No	No	Yes	Yes	Yes
Is there water in the schools?	Yes	Yes	No	No	Yes	No
If so, what type of water supply?	Rain water tank	Tap	N/A	N/A	Rain water tank	N/A
Does the water point function well?	Only if it rains	No	N/A	N/A	Only if it rains	N/A
Is there water available in the toilet facilities?	No	No	No	No	No	No
Are the girls’ toilets separate from the boys’ toilets?	Yes	Yes	Yes	Yes	Yes	Yes
Are the pupils’ toilets separate from the teachers’ toilets?	Yes	Yes	Yes	Partially	Partially	Yes
Are there locks on inside of cubicle doors?	Yes	No	No	No	No	No
Are there roofs on the toilets?	Yes	Yes	Yes	Yes	Yes	Yes
Are all the doors in place?	Yes	No	No	No	Yes	No
Are there toilets for girls with disabilities?	Yes	No	Yes	Yes	No	No
Are the toilets for girls with disabilities accessible for them?	No	N/A	No	No	N/A	N/A
Is there a dustbin available in toilet facilities to dispose of used sanitary products?	No	No	No	No	No	No
Is there an incinerator in school for menstrual materials?	No	No	No	No	No	No

### Focus group discussions (FGDs)

There were three main themes that were discussed about menstruation in the FGDs: the taboo nature of menstruation, the challenges facing schoolgirls and their recommendations for solutions to these challenges. These are described below.

**Taboo nature of menstruation:** The taboo nature of menstruation manifested itself as the girls struggled to discuss menstruation. Their nervous laughs, avoidance of eye contact, and the fact that they often turned their faces towards the floor when speaking drew attention to the fact that menstruation is a shameful and embarrassing experience and topic of conversation, even in a private, confidential, female-only environment. This may be partly attributed to the fact that the FGDs were led by researchers that the girls had only recently met.

**Challenges facing schoolgirls during menstruation:** Participants disclosed that they had missed school for menstrual-related reasons. Key challenges that girls identified were the lack of adequate sanitary materials, school sanitary facilities where they could change and wash, and pain relief. Girls who attended school during menstruation said they struggled to concentrate in lessons and did not want to participate in class activities for fear that others might recognise their menstrual status if their menstrual cloth fell out or if their menstrual blood stained their clothes.

**Girls’ recommendations for solutions:** For effective MHM, girls recommended that NGOs provide them with effective menstrual materials, adequate sanitary facilities in schools, MHM education and pain relief. Key features the girls recommended for their ideal toilets were: privacy (functioning cubicle doors and a roof); a room in the toilet block with water, soap, a basin and toilet paper for them to clean themselves and their menstrual materials; better lighting so they can see when they clean and change their menstrual materials and check for stains; and the provision of more toilet cubicles for girls. Whilst some FGD participants recommended a place to dispose of menstrual products, most girls overlooked this and envisaged throwing them in the pit latrines.

### Interviews

Key themes that emerged from the interviews were the challenges schoolgirls and female teachers face during menstruation, the sense of shame surrounding menstruation, and male headteachers’ lack of interest in and knowledge of menstruation. All interviewees affirmed that girls often miss school during menstruation. Key reasons given were the lack of adequate school sanitary facilities where girls could wash and change, sanitary materials (as commercial sanitary pads were too expensive), and painkillers for menstrual pain.

Head teachers and female teachers interviewed reported that girls missed school during menstruation to avoid the shame and embarrassment of menstrual accidents, whether this be due to menstrual blood leaking and staining their uniform or a menstrual cloth falling out. A senior woman teacher said that female teachers also sometimes miss school for menstrual-related reasons, for example when their menstrual materials do not absorb the blood sufficiently. Girls’ fear of dropping their menstrual cloth if they are beaten at school (a concern also expressed by the girls in the FGDs) was a further reason reported for menstrual-related absenteeism. In addition, interviewees explained that girls are often too ashamed to ask their parents for soap to wash during menstruation and consequently have to hide leftover soap after cleaning the family clothes so that they can wash their menstrual cloth.

Male headteachers were rarely able to go into much detail in the interviews as they lacked knowledge about MHM. For example, when asked what the government should do to support the girls with MHM, one head teacher replied that they should provide ‘special rooms with all the equipment that is needed for ladies’. When the interviewer probed further he could not give further details. Another headteacher gave a similar response: ‘All that is wanted. There are very many. Pads, cotton wool. Even some I do not know because I am not female’. Not only does this response highlight the head teacher's lack of knowledge about girls’ menstrual needs, but it also draws attention to his disinterest in the issue, as he perceives MHM as a female issue. This perspective was also evident as headteachers repeatedly emphasised that the girls’ MHM was the responsibility of the senior female teacher and they were not always aware of how senior female teachers assisted the girls or of what the school offered menstruating girls.

Senior female teachers reported that male headteachers rarely allocate sufficient funding for resources and facilities to help girls to manage their menstrual hygiene because they are not aware and/or not interested in girls’ menstrual needs. This is sometimes influenced by views in the wider community. One senior woman teacher reported an occasion when a headteacher had wanted to purchase sanitary products for schoolgirls but had been prevented from doing so as the local community did not think this was a suitable way for school money to be spent.

## Discussion

### Statement of principal findings

This study found that menstrual-related challenges pose a significant problem for girls’ education in rural government-run primary schools in the Rukungiri district of Uganda. We found that the majority of girls surveyed were at risk of menstrual-related absenteeism. This was supported by the findings of the FGDs and interviews.

### Strengths and weaknesses of this study

This study recognises the girls who took part as the experts of their own MHM experiences, asking them to identify the menstrual-related challenges they faced and to devise the solutions they would recommend to address these challenges. All questions in the questionnaire had a high response rate, therefore the results are highly representative of the respondents. The girls’ voiced experiences of MHM were recorded through a range of participatory methods in the FGDs.

We chose to collect self-reported data in this study in order to capture the voice of the girls whose menstrual hygiene practices we were exploring. However, self-reported data is limited due to the possibilities of recall bias and under-reporting [26, 27]. Recall bias may have led to girls under or over-estimating menstrual-related absenteeism as a result of selective memory. For example, they may have based their answers on their most recent menstrual experience which might have been unusually heavy. It is also likely that there was under-reporting as menstruation is a sensitive, taboo subject to discuss. Although the questionnaire and answer options were translated orally for the girls, the fact that the written version that the girls completed was in English may have affected their results. A further limitation was the fact that not all concepts could be translated into Rukiga. Such limitations may explain slight inaccuracies in certain results, such as girls reporting that they used tampons or menstrual cups when these products were not available or affordable on the local market.

### Strengths and weaknesses in relation to other studies

Although studies into the impact of menstruation on girls’ absenteeism often focus on secondary schools, and sometimes private schools, because they are more educated and articulate, this study focused on 13-16 year old girls at government-run primary schools in a rural, low-income setting. We chose this target population because, even though they are particularly at risk of poor MHM, this age-group of girls have not been included in discussions regarding MHM. As girls’ school dropout rate increases dramatically at secondary school level, focusing on primary schoolgirls meant that the study could capture the voices of girls who may have been overlooked by the majority of previous studies.

Whilst many studies about girls’ menstrual hygiene practices either employ qualitative or quantitative methods, this study combines qualitative methods with quantitative methods to provide a more detailed picture of the girls’ situation [16]. The interviews and FGDs provide a boarder context for the questionnaire responses. They also complement each other and therefore add robustness to the findings, thus offsetting the disadvantages of these methods when they are employed separately.

A limitation to this study in relation to other studies is that it potentially overlooks girls who may have been absent from school when we conducted the research or who had already dropped out of school for menstrual-related reasons. A further limitation is the study population size, which was smaller than some studies [28].

### Discussion of important differences in results

The incidence of girls who reported menstrual-related absenteeism in this study was lower than studies found in Malawi (100%) [12] and in the Chitwan district of Nepal (70.7%) [10] but much higher than the Dhading, Morang, Lalitpur and Kathmandu districts in Nepal (53%) [11], and in Kenya (50.2%) [14] and Uganda (14%) [25]. The mean number of days that girls reported missing school for menstrual concerns per month was also lower than the number reported in other countries such as Malawi (1-3 days) [12], Kenya (1.66 days) [14], Uganda (3-5 days) [29, 30], Ghana (3-5 days) [31], and Timor Leste (3-4 days) [32]. Varying levels of menstrual-related absenteeism are to be expected, owing to the diversity of socio-economic settings where girls’ MHM practices have been examined. Girls from more socially deprived backgrounds are more likely to miss school during menstruation as they are less likely to have what they need for good MHM.

The most cited reason for menstrual-related absenteeism was the lack of a place for the girls to wash and change at school and this was confirmed by the toilet assessment. Toilets in each school were found to be unclean and lacked soap, water, sufficient light, and privacy, amongst other important factors for MHM. The deficiencies of the toilets for menstruating girls were further demonstrated from the FGDs when girls were asked to design their ideal school toilets. The lack of suitable school toilets for menstruating girls has also been a common theme in previous studies [12, 13, 20].

The additional reasons for girls missing school during menstruation that girls reported (fear of staining clothes, menstrual pain and a lack of sanitary pads) have also been documented in other studies about menstrual-related absenteeism [12, 13, 33–35]. This demonstrates that they are common obstacles to girls’ school attendance and should form the focus of future interventions. The link between corporal punishment and menstrual-related absenteeism that girls mentioned in the FGDs has been absent from previous studies. This may be because girls participating in other MHM studies may not have considered this a reason for menstrual-related absenteeism or felt safe enough to share this information. Alternatively, previously, they may not have been given the opportunity to share additional reasons for menstrual-related absenteeism.

These findings highlight the harmful effects of poor menstrual hygiene management on girls’ education. It causes girls to miss sections of the school syllabus, which are difficult to catch up due to the small amount of academic support available and to risk missing important exams [11, 30]. This puts them at a further disadvantage to boys and may increase the likelihood that they will drop out of school. This can have detrimental effects to their future prospects because pupils need to pass their Primary Leaving Exam (PLE) to secure a place at a free secondary school [36].

Female teachers sometimes also missed school because they struggled to manage their menstrual hygiene in the school environment and this highlights the fact that menstrual-related absenteeism is not just a risk for female pupils. The risk of female teachers having to miss school when they menstruate if they are not equipped for MHM has been highlighted previously [37, 38]. The menstrual-related absence of female teachers has damaging consequences for all pupils who risk missing out on several hours of education on a regular basis. In the context of the current global teacher shortage, this is a waste of valuable resources [39].

This study found that even if girls manage to attend school during menstruation, the fear of staining their clothes and being ridiculed reduces their concentration and makes them reluctant to participate in class. This concern has also been raised in other studies [13, 38].

The girls surveyed had poor knowledge of and incorrect beliefs about menstruation, which has also been found in studies in Tanzania, India and Malawi [20, 40]. Girls were aware that their understanding of menstruation was limited and in FGDs girls shared their desire for better MHM education. Female teachers had not received training about menstruation, which is likely to be a key factor that contributes to the girls’ lack of knowledge about menstruation.

Some FGD participants and interviewees recommended that a place to dispose of menstrual products should be provided, particularly if they were given disposable pads. However, most girls and teachers overlooked this and envisaged throwing them in the pit latrines. This reveals the need for any provision of menstrual materials to be combined with the establishment of disposal systems [12, 13] and education about sustainable disposal so that pit latrines do not become rapidly blocked, and thus out of order, as has occurred elsewhere [41]. The girls’ struggle to discuss menstruation highlights its taboo nature. This reflects the findings of other researchers who have conducted FGDs with girls on the topic of MHM, particularly in rural primary schools [13, 42, 43].

Male headteachers’ lack of knowledge about girls’ menstrual needs and their reluctance to engage with this issue, despite the fact that some recognised that it was a reason behind female pupils’ absenteeism, suggests that they did not consider MHM important. Consequently, they do no allocate sufficient resources to this area. This point has been overlooked in other studies.

### Meaning of the study

This study highlights the existence and damaging effect of poor MHM on girls’ education in the Rukungiri District of Uganda. It supports the findings of other studies into the MHM of girls in low-income settings, whilst introducing unexplored nuances to the discussion. These findings are highly significant because girls have human rights to education, health and water and sanitation. Furthermore, educating girls has broad benefits for a country, including its economic development and the population's overall health [44, 45].

### Unanswered questions and future research

Small-scale, local projects have estimated the extent of poor MHM and its impact on girls’ education in low-income settings. A larger-scale study would capture this more accurately, specifically with regards to dropout rates and reduction in qualifications.

## Conclusion

In many low and middle-income countries, girls lack accurate information about and resources and facilities for effective Menstrual Hygiene Management. Consequently, they are likely to miss school or to struggle to concentrate and participate in lessons when they are menstruating. Girls who are unable to manage their menstrual hygiene at school risk missing a substantial proportion of their education and falling behind, which could lead to them dropping out of school altogether. This has negative consequences, for the girls and others, because educating a girl has significant benefits for her family, community, and country [15]. A large-scale study would be able to quantify the extent and impact of poor Menstrual Hygiene Management on girls’ education and suggest feasible solutions to ensure poor MHM does not prevent girls from reaching their potential at school.
